# Supplementary material for: A Systematic Review on the Antimicrobial Properties of Mediterranean Wild Edible Plants: We Still Know Too Little about Them, but What We Do Know Makes Persistent Investigation Worthwhile
Source: Foods. 2021 Sep 18;10(9):2217. doi: 10.3390/foods10092217 (PMC8471169; doi:10.3390/foods10092217)
Supplement: Supplementary file 1 [file foods-10-02217-s001.zip › foods-1390647-supplementary.pdf]

Supplementary material

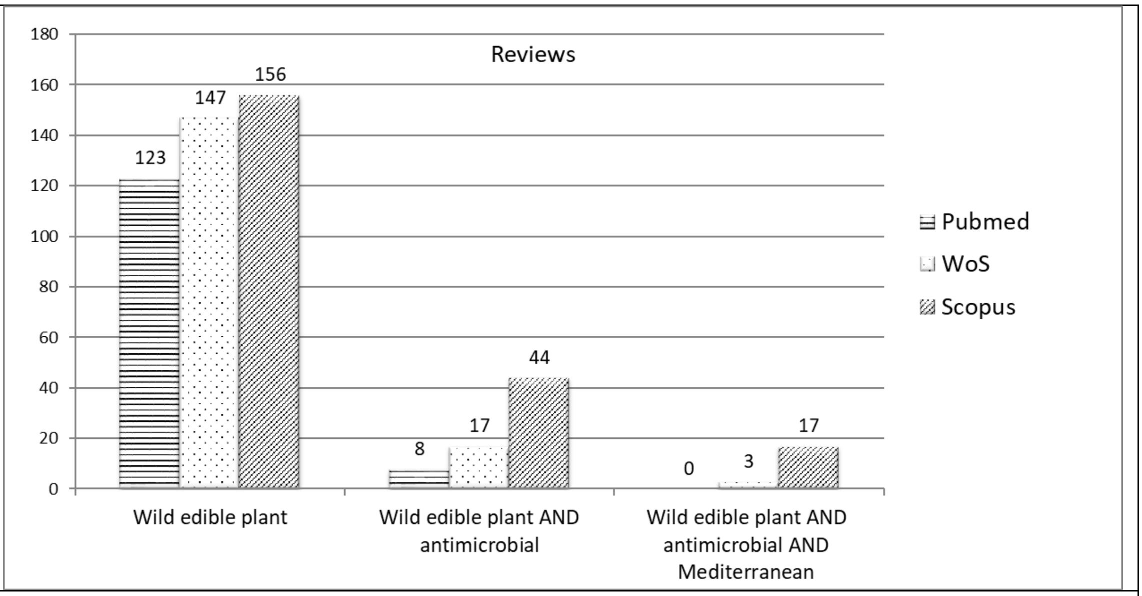

Figure S1. Initial survey on published reviews concerning Mediterranean Wild Edible Plants. Number of reviews retrieved by the three databases engine search, progressively filtered with the three selected keywods, whitout any time range and geographic filters.

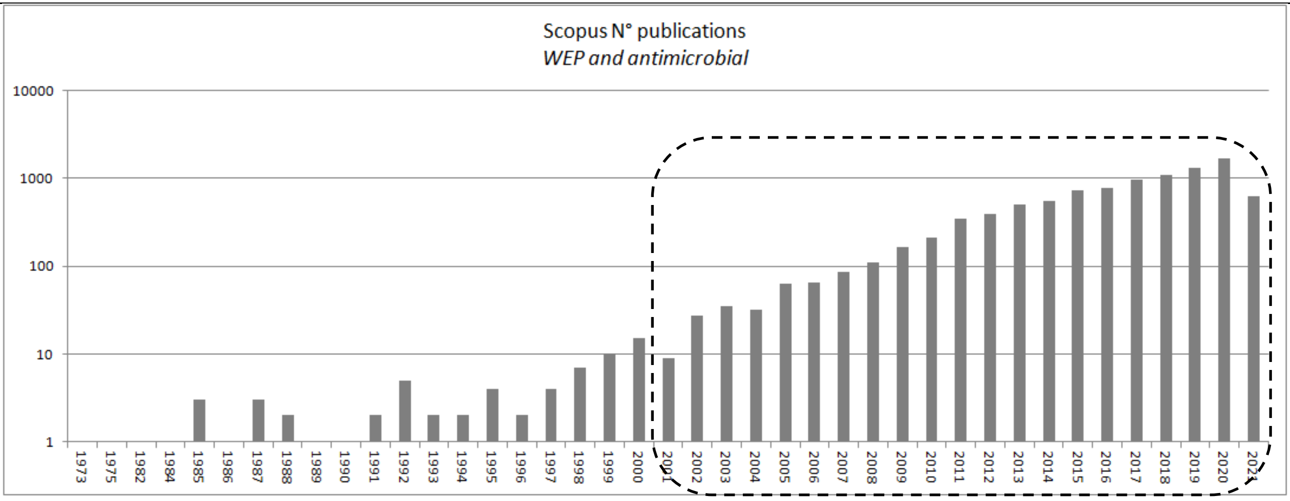

Figure S2. Selection of the time interval, according to the number of publications found with Wild Edible Plant (WEP) and antimicrobial keywords. We selected the last 20 years, in which the publication number grew significantly (from ten to thousand per year). Last search made on 14 April 2021.

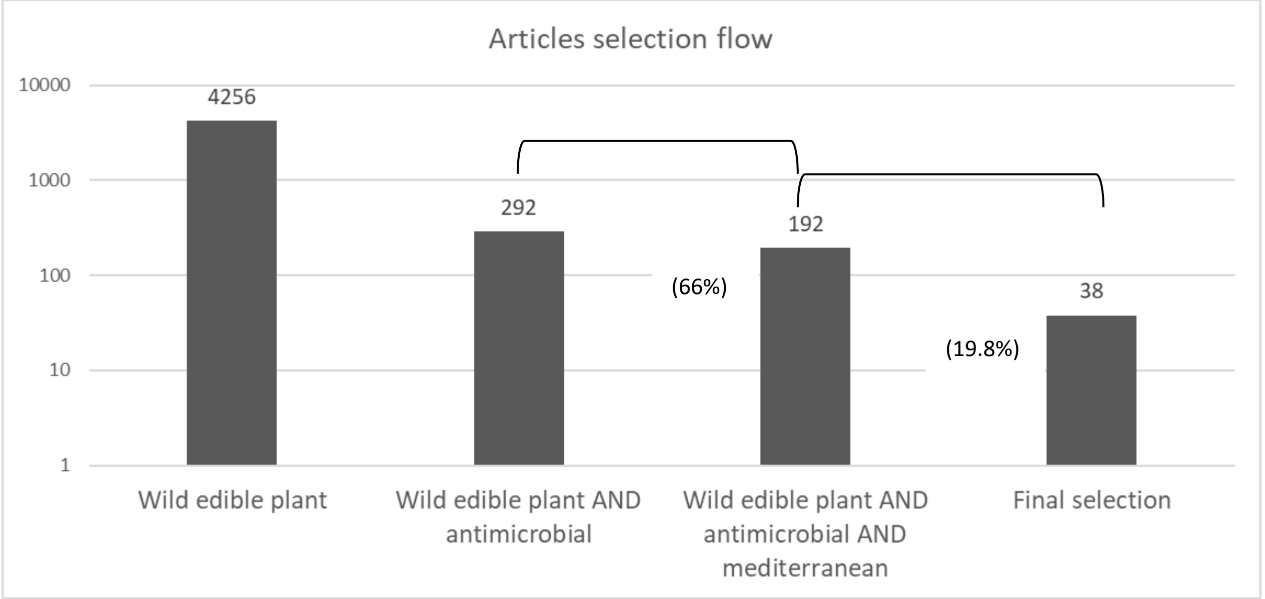

Figure S3. Articles selection flow, starting from the total matches obtained in the three database with the first keyword, Wild Edible Plants (WEPs), in the publication years range 2001-2021, and excluding USA, China, Japan and India countries of authors' affiliation in the Scopus database. The number of articles retrieved on antimicrobial Mediterranean WEPs (MWEPs) represents the 66% of the total articles on antimicrobial WEPs, and the final number of articles included in this review represent the 19.8% of MWEPs ones. Last search made on 14 April 2021.

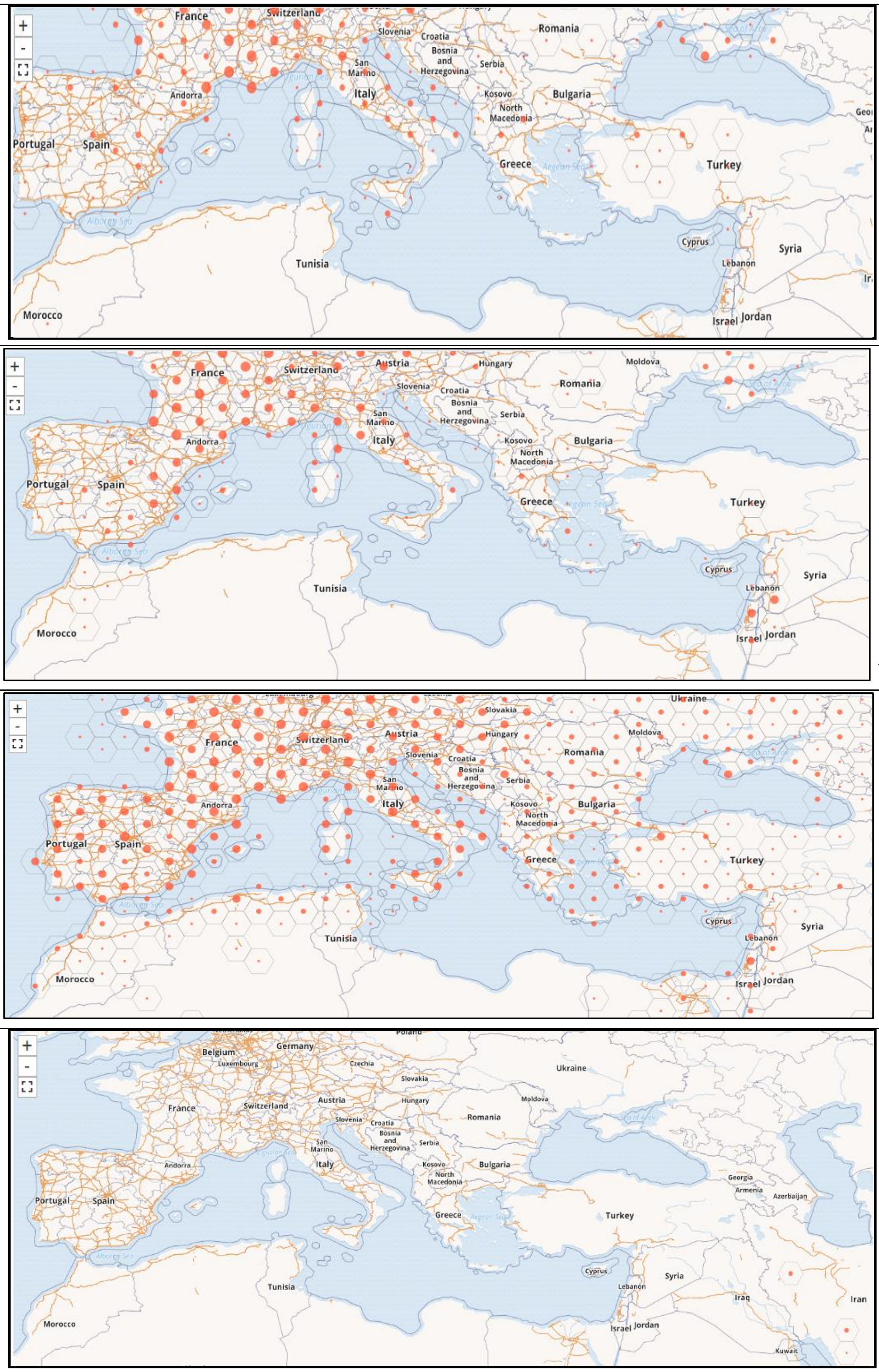

a.

b.

c.

d.

Figure S4. Examples of species geo-localization by search on GBIF | Global Biodiversity Information Facility homepage (https://www.gbif.org/species). Example of a. *Diplotaxis tenuifolia*, a plant species present in the Mediterranean basin; b. Taraxacum Genus, widely distributed in the Mediterranean basin; c. *Portulaca oleracea*, a plant species abundantly distributed in the Mediterranean basin; d. *Thymus daenesis*, a plant species totally absent in Mediterranean basin.

Species	Families	% of species	% of species	Grouped
14	**Asteraceae**	19	**69**	**8 Families 51 Species**
9	**Apiaceae**	12		
6	**Brassicaceae**	8		
6	**Caryophyllaceae**	8		
6	**Lamiaceae**	8		
4	**Fabaceae**	5		
3	**Polygonaceae**	4		
3	**Rutaceae**	4		
2	Amaranthaceae	3	**16**	**6 Families 12 Species**
2	Amaryllidaceae	3		
2	Malvaceae	3		
2	Myrtaceae	3		
2	Oleaceae	3		
2	Plantagineceae	3		
1	Apocynaceae	1	**15**	**11 Families 11 Species**
1	Araceae	1		
1	Asphodelaceae	1		
1	Boraginaceae	1		
1	Crassulaceae	1		
1	Euphorbiaceae	1		
1	Orobanchaceae	1		
1	Papaveraceae	1		
1	Portulacaceae	1		
1	Rosaceae	1		
1	Urticaceae	1		
**74**	**25**	**100,0**	**100,0**	

Table S1. List of all the Families and Species described in the analysed studies. It is worth noting that the first 8 families accounted for 69% of the studied species (51 species out of 74).

Families with the most studied species	Species	N° publications	Ref.
Amaranthaceae	*C.album*	2	[11,44]
Amaranthaceae	*C.murale*	2	[29,41]
**Amaryllidaceae**	***A.roseum***	**4**	[21,27,32,33]
**Apiaceae**	***F.vulgare***	**3**	[24,29,31]
Asphodelaceae	*E.spectabilis*	2	[18,22]
**Asteraceae**	***C.raphanina***	**4**	[16,17,20,37]
Asteraceae	*C.coronarium*	2	[24,35]
Asteraceae	*C.pumilum*	2	[13,24]
Asteraceae	*S.asper*	2	[7,19]
**Asteraceae**	***S.oleraceus***	**4**	[7,8,13,19]
Asteraceae	*T.officinale*	2	[8,19]
Brassicaceae	*N.officinale*	2	[41,44]
Caryophyllaceae	*S.vulgaris*	2	[19,39]
Lamiaceae	*Z.clinopodioides*	2	[25,40]
Papaveraceae	*P.rhoeas*	2	[29,44]
Polygonaceae	*P.aviculare*	2	[30,44]

Table S2. List of the 16 most studied species (described in more than one publication), belonging to 10 out of the total 25 botanical families. Four species (in bold) are analysed in more than two studies each, accounting for 39.5% of the whole thirty-eight studies reviewed.

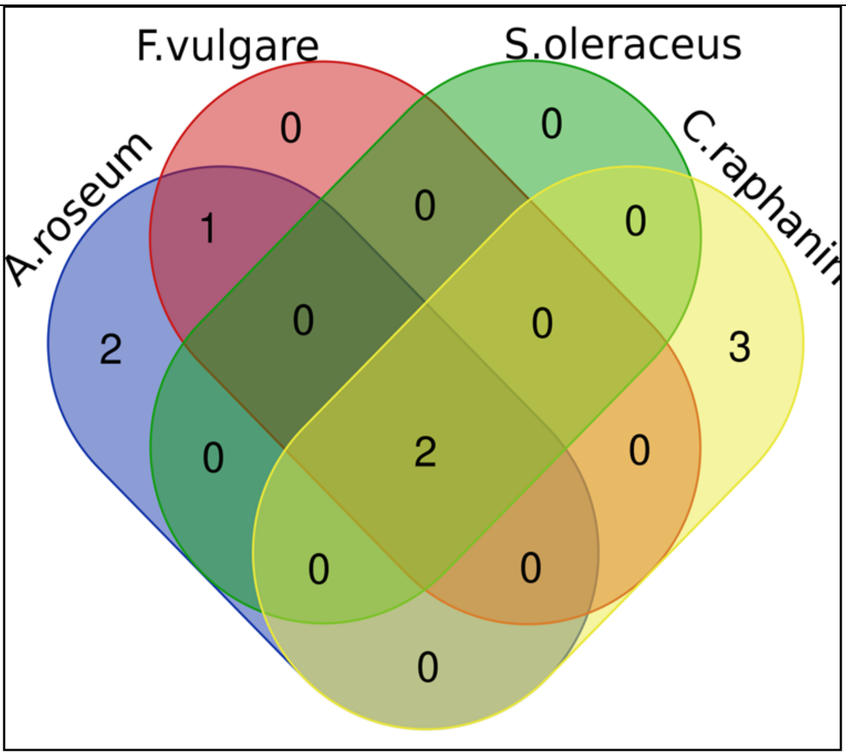

MWEPs Species	total	Bacterial species
Allium roseum ✚<br>*Centaurea raphanina* ✚<br>*Foeniculum vulgaris* ▬<br>*Sonchus oleraceus* ✚	2	*E.coli S.aureus*
Allium roseum ✚<br>*Foeniculum vulgaris* ▬	1	*P.aeruginosa*
Allium roseum ✚	2	*M.luteus S.epidermidis*
Centaurea raphanina ✚	3	✧ *E.cloacae B.cereus S.typhimurium*

http://bioinformatics.psb.ugent.be/cgi-bin/liste/Venn/calculate_venn.htpl

Figure S5. Venn Diagram of the common bacterial species used to assess their antimicrobial properties by the four MWEPs species analysed in more than two studies. The capacity to inhibit the bacterial growth is indicated with a plus symbol, the absence of inhibiting effect is indicated with a minus symbol, and a star symbol specify the case of a single bacterium sensible to the extract, as for the case of *C.raphanina* inhibiting only *E.cloacae* and not *B.cereus* and *S.typhimurium*.
In the case of *F.vulgaris,* the only bacterium sensible to its extract was the Gram-positive *Staphylococcus mutans,* not assayed with the other MWEPs species.

Species	Disk diff.	MIC mg/ml	MBC mg/ml	IC_{50}	Bacteria	MFC	Fungi	Extract	Ref
Sonchus oleraceus<br>***Sonchus arvensis***<br>***Sonchus asper***<br>***Sonchus uliginosus***	√	√			Gram-negative:<br>***Escherichia coli***<br>***Salmonella enterica***<br>*Vibrio parahaemolyticus*<br>Gram-positive:<br>***Staphylococcus aureus***			Methanolic of aerial parts air-dried, pulverized and stored at -48°C.	[7]
Reicardia picroides<br>***Picris echioides***<br>*Urospermum picroides*<br>***Taraxacum officinale***<br>*Hymenonema graecum*<br>***Sonchus oleraceus***<br>***Hedypnois cretica***<br>***Taraxacum spp***		√	√		Gram-negative:<br>***Salmonella typhimurium***<br>***E. coli***<br>*Enterobacter cloacae*<br>Gram-positive:<br>***Bacillus cereus***<br>***S. aureus***<br>***Listeria monocytogenes***	√	***Penicillium ochrochloron***<br>***Penicillum funiculosum***<br>***Penicillum verrucosum* var. *cyclopium***<br>*Aspergillus fumigatus*<br>***Aspergillus ochraceus***<br>*Aspergillus niger*	Hydromethanolic using a 30 g/L solid/liquid ratio with methanol/water (at 25 °C at 150 rpm). Plants cultivated from wild seeds, fresh parts frozen and lyophilized.	[8]
Ononis natrix	√				Gram-negative:<br>*S. typhimurium*<br>*E. coli*<br>*Pseudomonas aeruginosa*<br>Gram-positive:<br>*S. aureus*<br>***Enterococcus faecalis***	√	*Candida albicans*	Methanolic extract of dried leaves.	[9]

Raphanus raphanistrum		√	√		Gram-negative:<br>*E. coli*<br>*Klebsiella pneumoniae*<br>*Morganela morganii*<br>*Proteus mirabilis*<br>*P. aeruginosa*<br>Gram-positive:<br>*E. faecalis*<br>*L. monocytogenes*<br>Meth-resistant *S.aureus* (MRSA)			Fresh and lyophilized leaves decocted in boiling water and extracted with 80% ethanol/water.	[10]
Bidens pilosa, Chenopodium album		√			Gram-negative:<br>***E. coli***<br>*P. aeruginosa*<br>*Salmonella pooni*<br>*Serratia marcescens*<br>*K. pneumoniae*<br>Gram-positive:<br>***B. cereus***<br>*S. epidermidis*<br>*S. aureus*<br>*Micrococcus kristinae*<br>***Streptococcus pyogenes***			Acetone, Methanol, Water extracts of fresh and air-dried leaves.	[11]
Heracleum pyrenaicum* subsp. *orsinii		√	√		Gram-negative:<br>***E. coli***<br>***P. aeruginosa***<br>***S. typhimurium***<br>*E. cloacae*<br>Gram-positive:<br>***B. cereus***<br>***S. aureus***<br>*L. monocytogenes*<br>*Micrococcus flavus*	√	*A .niger*<br>***A. fumigatus***<br>***Aspergillus versicolor***<br>***A. ochraceus***<br>***Trichoderma viride***<br>***P. funiculosum***<br>*P. ochrochloron*<br>*P. verrucosum*<br>*P. verrucosum* var. *cyclopium*	Air-dried material powdered or crashed and hydrodistilled. Collecting solvent was n-hexane. The oils dried over anhydrous sodium sulfate and kept at 4 °C.	[12[

Sonchus oleraceus<br>*Cichorium pumilum*<br>***Portulaca oleracea***	√					√	***Aspergillus flavus***<br>***A. ochraceus***<br>***Aspergillus parasiticus***	Fresh leaves dried in an oven, ground to a fine powder. Powder plants extracted by distilled water at room temperature for 24 h, then centrifuged and evaporated to near dryness. The resulting viscous powder dissolved to obtain stock solution.	[13]
Psidium cattleianum<br>*Psidium guajava*	√				Gram-negative:<br>*E. coli*<br>*P. aeruginosa*<br>Gram-positive:<br>*S. aureus*<br>*Bacillus subtilis*			Fresh, fully ripe fruits lyophilized to dried material. A portion extracted successively with hexane, ethyl acetate and methanol.	[14]
Scandix pecten-veneris	√				Gram-negative:<br>***E. coli***<br>*P. aeruginosa*<br>***S. typhi***<br>***Proteus vulgaris***<br>Gram-positive:<br>***S. aureus***<br>***B. subtilis***<br>***Streptococcus pneumoniae***	√	***A. flavus***<br>***A. niger***<br>*A. parasiticus,*<br>***C. albicans***	Fresh and healthy leaves dried in the shade for at least two weeks, then crushed and stored in the dark. Extraction by soaking dry powdered leaves in methanol.	[15]
Centaurea raphanina		√	√		Gram-negative:<br>***E. coli***<br>*S. typhimurium*<br>*E. cloacae*<br>Gram-positive:<br>*S. aureus*<br>***B. cereus***	√	***A. niger***<br>***A. fumigatus***<br>***A. versicolor***<br>***Trichoderma viride***<br>***P. funiculosum***<br>***P. verrucosum* var. *cyclopium***	Fresh leaves put in plastic food bags and stored in freezing conditions. Then, frozen leaves lyophilized, ground to powder, and stored in deep-freezing conditions. Solvent employed for antimicrobial assays not specified	[16]

Centaurea raphanina		√	√		Gram-negative:<br>***E. coli***<br>*S. typhimurium*<br>*E. cloacae*<br>Gram-positive:<br>***S. aureus***<br>***B. cereus***	√	***A. niger***<br>***A. fumigatus***<br>***A. versicolor***<br>***T. viride***<br>***P. funiculosum***<br>***P. verrucosum* var.**<br>***cyclopium***	Fresh leaves put at −80 °C until lyophilization, ground to powder, and stored again at −80 °C.<br>Hydroethanolic extracts of the samples by stirring the dried plant material with ethanol-water for 60 min.	[17]
Eremurus spectabilis	√				Gram-negative:<br>*Aeromonas hydrophila*<br>***E. coli***<br>***K. pneumoniae***<br>***P. aeruginosa***<br>*S. typhimurium*<br>*Yersinia enterocolitica*<br>Gram-positive:<br>***S. aureus***<br>*Bacillus brevis*<br>*B. cereus*<br>*B. subtilis*<br>***L. monocytogenes***			Leaves and roots air dried at room temperature used for extracts. Acetone used in an amber flask. All extraction solvents evaporated under reduced pressure. Stored in dark at 4°C.	[18]
Borago officinalis<br>***Orobanche crenata***<br>***Plantago coronopus***<br>***Plantago lanceolata***<br>***Sanguisorba minor***<br>*Silene vulgaris*<br>***Sonchus asper***<br>***Sonchus oleraceus***<br>*Taraxacum officinale*						√	***Botrytis cinerea***<br>***Monilinia laxa***<br>***Penicillium digitatum***<br>***P. expansum***<br>***P. italicum***<br>*Aspergillus carbonarius*<br>***A. niger***	Species dried in a ventilated oven, finely ground in a grinder to obtain a dry powder, and stored under vacuum in a cool room. An amount of dry powder extracted twice with refluxing aqueous methanol. After extraction, the methanolic extracts filtered and evaporated to dryness under reduced pressure at 35 ◦C.	[19]
Centaurea raphanina						√	***A. niger***<br>*A. flavus*<br>***A. versicolor***<br>***A. ochraceus***<br>*T. viride*<br>*P. funiculosum*	Fresh plant material finely ground and extracted at room temperature with cyclohexane-Et_2OMeOH. The extract washed with brine, the aqueous layer re-extracted with EtOAc, and the organic layer	[20]

							P. ochrochloron<br>***Cladosporium cladosporioides***<br>***Alternaria alternata***	dried with Na_2SO_4 and concentrated under reduced pressure.	
Allium roseum	√	√	√		Gram-negative:<br>***E. coli***<br>***P. aeruginosa***<br>***S. typhimurium***<br>Gram-positive:<br>***S. aureus***<br>***S. epidermidis***<br>***B. subtilis***<br>*B. cereus*<br>***Micrococcus luteus***<br>***E. faecalis***	√	***C. albicans***	Separated stems, bulbs and flowers cleaned, washed, cut into small pieces, and lyophilized. Each sample extracted separately with either cold acetone/water, methanol/water, or distilled water. Extracts centrifuged and supernatants concentrated using a rotary evaporation under vacuum. Extracts immediately used or stored at −20◦C.	[21]
Eremurus spectabilis	√				Gram-negative:<br>*E. coli*<br>Gram-positive:<br>*S. aureus*<br>*B. subtilis*<br>*L. monocytogenes*	√	*Saccharomyces cerevisiae*	Leaves and roots air dried at room temperature. Acetone used in an amber flask. Extract mixed by a magnetic stirrer. Mixture left at room temperature for 24 h. Extract filtered to obtain particle free extract. Residue re-extracted twice with acetone and filtered. All extraction solvents evaporated under reduced pressure. Stored in dark at 4°C.	[22]
Ruta angustifolia		√	√		Gram-negative:<br>*E. coli*<br>*P. aeruginosa*<br>*S. enteritidis*<br>*Enterobacter aerogenes*<br>Gram-positive:<br>*S. aureus*<br>*B. cereus*<br>*L. monocytogenes*	√	*C. albicans*<br>*A. niger*	Plant material reduced to a fine powder and extracted with ethanol or methanol by percolation. Ethanolic and methanolic extracts of wild growing plants obtained after evaporation to the dryness under reduced pressure below 40 ºC.	[23]

Foeniculum vulgare<br>***Salvia palaestina***<br>***Micromeria fruticose***<br>***Trigonella foenum-graecum***<br>***Cichorium pumilum***<br>***Salvia hierosolymitana***<br>***Ruta chalepensis***<br>***Chrysanthemum coronarium***				√	Gram-positive:<br>***Staphylococcus mutans***			Specimens dried in the shade for one month. Ground up and packed in the tubes of Soxhlet. A mixture of solvents (water, ethanol, ethyl acetate, and hexane) introduced. The solvents from each extract evaporated in an oven under reduced pressure. The polar extracts dissolved in water, while the non-polar extracts dissolved in dimethyl sulfoxide (DMSO).	[24]
Ziziphora clinopodioides	√	√	√		Gram-negative:<br>***E. coli***<br>***K. pneumoniae***<br>*P. aeruginosa*<br>***Proteus vulgaris***<br>***Citrobacter freundii***<br>Gram-positive:<br>***S. aureus***<br>***L. monocytogenes***<br>***S. epidermidis* (coag. Neg)**<br>***S. saprophyticus* (coag. Neg)**			Leaves dried in shadow and crushed by mill. Each sample soaked in methanol and 48 hours later smoothed by filter paper. Extracts obtained using rotary machine, concentrated and dried at the same temperature for 2 days and gradually dried. For production of oil, water distillation method applied.	[25]
Umbilicus rupestris		√	√		Gram-negative:<br>*E. coli*<br>*K. pneumoniae*<br>*M. morganii*<br>*Proteus mirabilis*<br>*P. aeruginosa*<br>Gram-positive:<br>*E. faecalis*<br>*L. monocytogenes*<br>MRSA			Leaves lyophilized and reduced to a fine powder. Hydroethanolic extracts prepared by extracting freeze-dried sample with an ethanol:water solution. After filtration, the plant residue re-extracted and the combined filtrates evaporated under pressure at 40 °C and lyophilized.<br>Decoctions prepared using freeze-dried samples and heated distilled water. The mixture boiled using a heating plate and then filtrated. The obtained decoctions frozen and lyophilized to obtain a dried extract.	[26]

Allium roseum	√				Gram-negative:<br>*E. coli* ATCC 25922<br>*P. aeruginosa* ATCC 27853<br>Gram-positive:<br>*S. aureus* ATCC25923<br>*S. epidermidis* CIP106510<br>*M. luteus* NCIMB 8166			Leaves air-dried in shade, blended into fine powder and stored in a dry place and at dark.<br>**Aqueous Extracts.**<br><u>Macerate</u>: plant powder macerated in water. Filtered then the total filtrate lyophilized.<br><u>Digestion</u>. Dried powdered leaves extracted with water at 50°C then filtered and lyophilized.<br><u>Decoction</u>. Prepared by boiling the powdered leaves in water. After filtration, the extract lyophilized.<br><u>Infusion</u>. Powdered leaves held in boiling water. After filtration, the liquid phase frozen and lyophilized.<br>**Organic Extracts**. Powdered leaves homogenized in petroleum ether. The final residue extracted again with three solvents: dichloromethane, methanol and then ethanol 80%. Then evaporated to dryness. The total organic residue dried and then further extracted with distilled water to obtain an exhausted organic extract. Finally, the exhausted residue decocted in water.	[27]
Origanum syriacum		√			Gram-negative:<br>*E. coli* ATCC 25922<br>*P. aeruginosa* ATCC 27853<br>Gram-positive:<br>***S. aureus* ATCC 25923**<br>MRSA<br>***Enterococcus faecium* ATCC 700221**			Leaves separated from the stems, washed and dried in the shade at room temperature. The EOs of the four samples of plant extracted by using a microwave-ultrasonic method. The obtained EO was collected into a clean beaker, dried over anhydrous sodium sulfate (Na2SO4) and stored in the refrigerator at 2–8 ° C.	[28]

Mercurialis annua<br>*Papaver rhoeas*<br>*Foeniculum vulgare*<br>*Chenopodium murale*<br>*Scolymus hispanicus*	√				Gram-negative:<br>*E. coli* CIP54127<br>*P. aeruginosa*<br>Gram-positive:<br>*S. aureus* CIP 209 ATCC 25923<br>*E. faecalis* ATCC19433	√	*Cryptococcus neoformans* CIP 960<br>*C. albicans*	Fresh plant material dried in an oven, and the powder material weighed. Powder is soaked for 48 hours in a mixture of two solvents, a polar solvent (ethanol) and an apolar solvent (dichloromethane). The mixture filtered, and the filtrate concentrated under reduced pressure using a rotary evaporator and the crude extract dried. The extracts stored at 4°C.	[29]
Sinapis arvensis,<br>*Polygonum aviculare*<br>*Tragopogon aureus*	√				Gram-negative:<br>*Agrobacterium tumefaciens*<br>***P. aeruginosa***<br>*Pseudomonas corrugate*<br>*Pseudomonas syringae* pv. *tomato*<br>*Yersinia frederiksenii*<br>*Yersinia pseudotuberculosis*<br>*Yersinia enterocolita*<br>*S. typhimurium*<br>*Serratia liquefaciens*<br>*Vibrio cholerae*<br>Gram-positive:<br>*B. cereus,*<br>*Corynebacterium diphtheriae*<br>Proteobacteria:<br>*Xanthomonas compestris*			Leaves separated from plants and dried at 50ºC in an oven, then dried leaves ground to a fine powder with a mortar and pestle and kept at room temperature prior to extraction for antioxidant activity and total phenolics analysis.	[30]
Foeniculum vulgare	√	√	√		Gram-negative:<br>*P. aeruginosa* CIP 82118<br>*S. enterica* CIP 8039<br>*E. coli* CIP 53126<br>Gram-positive:<br>*S. aureus* CIP 53156<br>*B. subtilis* CIP 5262<br>*M. luteus* CIP 5345	√	*A. niger* ATCC 16404<br>*C. albicans* ATCC 10231	Dried seeds were hydrodistilled using a Clevenger-type apparatus. Essential oil dried over anhydrous sodium sulfate and stored in dark vials at 48°C.	[31]

Allium roseum	√	√	√		Gram-negative:<br>*E. coli*<br>*P. aeruginosa*<br>*S. typhimurium*<br>Gram-positive:<br>*S. aureus*<br>*S. epidermidis*<br>*B. cereus*<br>*B. subtilis*<br>*M. luteus*	√	*C. albicans*	Plant extracted by three methods.<br>Method 1: powdering each sample with a mortar and pestle, stirring the powder in 0.05 M sulfuric acid, neutralizing the suspension with NaOH and removing the insoluble material by centrifugation and microfiltration.<br>Method 2: material homogenized with 1 M Tris HCl buffer (pH 8.8) filtered and centrifuged.<br>Method 3: each sample homogenized in 0.02 M phosphate buffer (pH 7.2) containing 0.1 M NaCl, stirred overnight, filtered, adjusted to pH 4.0 with acetic acid (50%, v/v), stirred and centrifuged. All extraction steps done at 4°C. Plant extracts stored at -20°C.	[32]
Allium roseum	√	√	√		Gram-negative:<br>***S. typhimurium* NRRLB 4420**<br>***E. coli* ATCC 25922**<br>***P. aeruginosa* ATCC 27853**<br>Gram-positive:<br>***S. aureus* ATCC 25923**<br>*S. epidermidis* CIP 106510<br>*M. luteus* NCIMB 8166<br>***B. cereus* ATCC 11778**<br>***B. subtilis* ATCC 168**<br>***E. faecalis* ATCC 29212**	√	***C. albicans* ATCC 1405**	Plant extracted separately by three methods. Method 1: powdering each sample with a mortar and pestle, stirring the powder in 0.05 M sulfuric acid, neutralizing the suspension with NaOH and removing the insoluble material by centrifugation and microfiltration.<br>Method 2: material homogenized with 1 M Tris HCl buffer (pH 8.8) filtered and centrifuged.<br>Method 3: each sample homogenized in 0.02 M phosphate buffer (pH 7.2) containing 0.1 M NaCl, stirred overnight, filtered, adjusted to pH 4.0 with acetic acid (50%, v/v), stirred and then centrifuged. All extraction steps done at 4°C. Plant extracts stored at -20°C.	[33]

Olea europeae<br>(cultivated)<br>*Olea ferrugineae*<br>(wild)	√				Gram-negative:<br>*E. coli* ATCC 25922<br>*P. aeruginosa* ATCC 27853<br>*K. pneumoniae* ATCC 15380<br>*S. typhimurium* ATCC 14028<br>Gram-positive:<br>*S. aureus* ATCC 6538<br>*L. monocytogenes* ATCC 13932<br>*B. cereus* ATCC 11778<br>*B. subtilis* ATCC 19659<br>*E. faecalis* ATCC 49452			Leaves crude extraction: fine powder of leaves dissolved separately using different organic solvents. Rotary flash evaporator used to concentrate all the extracts. Concentrated extract weighed and preserved in airtight bottles at 4°. For antibacterial assay, 15 mg of each extract dissolved in 1ml of DMSO as a solvent.<br>Crude oils extraction: Olive oils extracted from the ripe and unripe fruit by mechanical pressing and using n-hexane as a solvent.	[34]
Chrysanthemum coronarium<br>(or *Glebionis coronaria*)		√	√		Gram-negative:<br>*E. coli* UCMB-906<br>*P. aeruginosa* UCMB-900<br>Gram-positive:<br>***S. aureus* UCMB-904**	√	***C. albicans* UCMY-1918**	Samples for microbiological studies collected during their blossom stage. The extract of the above-ground part obtained by infusing air-dry material in 40% ethanol. Essential Oil by Clevenger method.	[35]
Allium macrochaetum		√			Gram-negative:<br>*E. coli* ATCC 25922<br>*K. pneumoniae* ATCC 4352<br>*P. mirabilis* ATCC 14153<br>*P. aeruginosa* ATCC 27853<br>Gram-positive:<br>*S. aureus* ATCC 29213<br>***S. epidermidis* ATCC 12228**<br>***E. faecalis* ATCC 29212**	√	*C. albicans* ATCC 10231,<br>*C. parapsilosis* ATCC 22019<br>*C. tropicalis* ATCC 750	Essential oil: Clevenger apparatus used for obtaining essential oil of bulb with water. EO dried over anhydrous sodium sulfate and diluted by dichloromethane.<br>Petroleum ether: The oil of bulbs obtained using Soxhlet apparatus with petroleum ether.<br>Water and ethanol extracts: samples homogenized separately in ethanol and water using a waring blender at high speed. The extracts filtered through cheesecloth, and<br>the residue re-extracted under the same condition with same solvents. The combined filtrate concentrated under vacuum at 35°C to dryness and lyophilized at −80°C. The extracts storaged at −20°C. Dry extracts diluted and filtrated.	[36]

Centaurea raphanina		√	√		Gram-negative:<br>*E. coli* ATCC 25922<br>*S. typhimurium* ATCC 13311<br>*E. cloacae* ATCC 35030<br>Gram-positive:<br>*S. aureus* ATCC 11632<br>*B. cereus* food isolate	√	***A. fumigatus* ATCC 9197**<br>*A. niger* ATCC 6275<br>*A. versicolor* ATCC 11730<br>***P. funiculosum* ATCC** 36839<br>***P. verrucosum* var. *cyclopium* food isolate**<br>***T. viride* IAM 5061**	Frozen leaves lyophilized, ground to powder, and stored in deep-freezing conditions.<br>The lyophilized plant material used to prepare hydroethanolic extracts, obtained by stirring the powder with of ethanol/water and filtered. The residue then re-extracted with additional hydroalcoholic mixture. The combined extracts concentrated at 40 °C under reduced pressure and further lyophilized.	[37]
Polygonum hydropiper	√				Gram-negative:<br>*E. coli*<br>*K. pneumoniae*<br>*M. morganii*<br>*Haemophilus influenzae*<br>Gram-positive:<br>*S. aureus*			Fresh leaves and stem separated, washed and air-dried at 25 ° C for 30 days in an air flux drying oven. The plant parts crushed to fine powder. Powdered samples placed in sterile sealed bags each and kept at 4 °C for further analyses. Extracts made with Ethanol, Acetone, Methanol, n-hexane, Chloroform and water.	[38]
S. alba<br>***S. conoidea***<br>***S. dichotoma***<br>***S. italica***<br>***S. supina***<br>***S. vulgaris***		√	√		Gram-negative:<br>***E. coli* ATCC 35210**<br>***P. aeruginosa* ATCC 27853**<br>***S. typhimurium* ATCC 13311**<br>*E. cloacae*<br>Gram-positive:<br>***L. monocytogenes* NCTC 7973**<br>***E. faecalis* hu- isolate**<br>***B. cereus* clinical isolate**<br>***M. flavus* ATCC 10240**<br>***S. aureus* ATCC 6538**	√	***A. versicolor* ATCC 11730**<br>***A. fumigatus* plant isolate**<br>***A. ochraceus* ATCC 12066**<br>***A. niger* ATCC 6275**<br>***P. ochrochloron* ATCC 9112**<br>***P. funiculosum* ATCC 36839**<br>***P. verrucosum* food isolate**<br>***T. viride* IAM 5061**	Aerial parts allowed to air dry for 10 days at room temperature. Dried plant samples ground to a fine powder. Powdered plant material extracted with methanol. The extracts filtered and concentrated under vacuum at 40 °C. The extracts stored at +4 °C in dark.	[39]

Ziziphora clinopodioides		√	√		Gram-negative:<br>*P. aeruginosa* ATCC 27853<br>*E. coli* ATCC 25922<br>*K. pneumoniae* ATCC 4352<br>*P. mirabilis* ATCC 14153<br>Gram-positive:<br>*S. aureus* ATCC 29213<br>***S. epidermidis* ATCC 12228**<br>*E. faecalis* ATCC 29212	√	*C. albicans ATCC 10231*<br>***C. parapsilosis* ATCC 22019**<br>***C. tropicalis* ATCC 750**	Samples macerated in ethanol for 24 hours at room temperature. The extract filtered and the residue re-macerated under the same condition with ethanol two more times. The combined filtrate concentrated in a vacuum at 35ºC to remove the organic solvent. The extract stored at -20ºC.	[40]
Chenopodium murale<br>***Eruca sativa***<br>***Malcolmia africana***<br>***Malva neglecta***<br>***Medicago polymorpha***<br>***Melilotus officinalis***<br>***Nasturtium officinale***	√				Gram-negative:<br>***E. coli***<br>***S. typhi***<br>***P. vulgaris***<br>Gram-positive:<br>***S. pneumoniae***			Non-plant materials and any visible dirt and insect parts removed from the plant samples. Plant leaves dried in shade, pulverized and stored in paper bags and analysed.<br>The plant material extracted with methanol by maceration and fractionated. The extract evaporated to dryness under vacuum and stored at 4°C.	[41]
Carissa macrocarpa		√	√		Gram-negative:<br>*E. coli* ESBL<br>*K. pneumoniae*<br>*K. pneumoniae* ESBL<br>*M. morganii*<br>*P. aeruginosa*<br>Gram-positive:<br>*S.aureus* MSSA<br>MRSA<br>*S. epidermidis*<br>*L. monocytogenes*<br>*E. faecalis*			Samples dried until at a constant weight in an incubator at 35°C. Then, plant material ground and the homogeneous samples stored in a desiccator protected from light. The hydroalcoholic extract obtained by maceration using aqueous ethanolic solution as the extraction solvent. After filtration, the solvent first evaporated at 40°C, under reduced pressure, in a rotary evaporator and the residual solvent removed in a freeze drier.	[42]

Smyrnium olusatrum<br>*Smyrnium perfoliatum*<br>*Smyrnium rotundifolium*<br>*Smyrnium cordifolium*<br>*Smyrnium connatum*<br>*Smyrnium creticum*	√				Gram-negative:<br>*E. coli* ATCC 39628<br>*E. cloacae* ATCC 13047D<br>*S. typhimurium* CCM5445<br>Gram-positive:<br>*Sarcina lutea* ATCC 9341NA	√	*C. albicans ATCC 10231*	Dried and powdered aerial parts reduced to coarse powder. Plant extracted with methanol at room temperature. The methanol evaporated to dryness after extraction progress. Sample solutions prepared by dissolving the extracts in dimethyl sulfoxide (DMSO).	[43]
Arum dioscoridis<br>***Chenopodium album***<br>***Malva sylvestris***<br>***Mentha longifolia* Huds.**<br>***Nasturtium officinale* Aiton,**<br>***Papaver rhoeas***<br>***Polygonum aviculare***<br>***Rumex acetosella***<br>***Sinapis alba***<br>***Urtica dioica***		√			Gram-negative:<br>***P. aeruginosa* ATCC 10145/isolated strain**<br>***Acinetobacter baumannii* RSKK 02026/ isolated strain**<br>***S. enteriditis* RSKK 538/ isolated strain**<br>Gram-positive:<br>***S. aureus* ATCC25923/ isolated strain**<br>***E. faecalis* ATCC 29212/ isolated strain**<br>***B. subtilis* ATCC 6633 / isolated strain**	√	***C. albicans* ATCC 10231**<br>***C. krusei* ATCC 6258**	Plants cleaned, dried at room temperature and powdered for subsequent extraction process.<br><u>Step 1 (non polar extract)</u>: Powdered plant parts macerated with n-hexane. After filtration, once more extraction performed on the residue. Then filtrated extracts combined and concentrated in vacuum at 40ºC. Extracts kept in the dark at +4ºC. The residue kept for the further methanol extraction.<br><u>Step 2 (polar extract)</u>: Residue obtained from hexane extraction macerated with 70% methanol by leaving them overnight. After extract filtration, once more extraction performed on the residue. Then filtrated extracts combined and concentrated in vacuo at 40ºC using a Rotary evaporator. Extracts kept in the dark at +4ºC until tested.	[44]

Table S3. Complete list of the botanical species studied, the extracts employed, the antimicrobial assays used, the bacteria and fungi species analysed. In bold we showed: 1. the MWEPs proved to be most effective; 2.the bacteria and/or fungi documented to be susceptible to MWEPs extracts.
